# Comparative Genomics, Infectivity and Cytopathogenicity of American Isolates of Zika Virus that Developed Persistent Infections in Human Embryonic Kidney (HEK293) Cells

**DOI:** 10.3390/ijms20123035

**Published:** 2019-06-21

**Authors:** Hebing Liu, Hsiao-Mei Liao, Bingjie Li, Shien Tsai, Guo-Chiuan Hung, Shyh-Ching Lo

**Affiliations:** Tissue Microbiology Laboratory, Division of Cellular and Gene Therapies, Office of Tissues and Advanced Therapies, Center for Biologics Evaluation and Research, Food and Drug Administration, Silver Spring, MD 20993, USA; hebing.liu@fda.hhs.gov (H.L.); hsiao-mei.liao@fda.hhs.gov (H.-M.L.); bingjie.li@fda.hhs.gov (B.L.); shien.tsai@gmail.com (S.T.); guo-chiuan.hung@fda.hhs.gov (G.-C.H.)

**Keywords:** Zika virus, viral persistence, HEK293, FLR strain, PRV strain

## Abstract

Zika virus (ZIKV) transmission can cause serious fetal neurological abnormalities. ZIKV persistence in various human cells and tissues can serve as infectious reservoirs and post serious threats to public health. The human embryonic kidney (HEK293) cell line with known neuronal developmental properties was readily infected by ZIKV in a strain-dependent fashion. Significant cytopathic effect in HEK293 cells infected by the prototype MR 766 strain of ZIKV resulted in complete loss of cells, while small numbers of HEK293 cells infected by contemporary ZIKV isolates (PRV or FLR strain) continued to survive and regrow to confluency in the culture around two months after initial infection. Most, if not all, of the cells in the two resulting persistently ZIKV-infected HEK293 cell lines tested positive for ZIKV antigen. Compared to HEK293 control cells, the persistently ZIKV-infected HEK293 cells had slower growth rates with some cells undergoing apoptosis in culture. The “persistent ZIKVs” produced constitutively by both PRV and FLR strains ZIKV-infected HEK293 cells had significantly attenuated cell infectivity and/or cytopathogenicity. Comparative genome sequence analyses between the persistent ZIKVs and the original inoculum ZIKVs showed no clonal selection with specific gene mutations in the prolonged process of establishing persistently PRV strain ZIKV-infected HEK293 cells; while selection of ZIKV subclones with mutations in the envelope, protein pr and multiple NS genes was evident in developing persistently FLR strain ZIKV-infected HEK293 cell line. Our study provides molecular insights into the complex interplays of ZIKV and human host cells in establishing ZIKV persistence.

## 1. Introduction

The emergence of Zika virus (ZIKV) in 1947 from Rhesus monkey in the Zika forest of Uganda has resulted in new challenges to global public health. Transmission of ZIKV in French Polynesia and South America in previously infection-naïve territories, has been associated with the development of pathologic neurological symptoms and central nervous system abnormalities in the human fetuses including microcephaly [1,2,3,4]. Viral persistence in the infected hosts has long been recognized as a common theme in flavivirus biology. Persistently flavivirus-infected cells hidden in tissues are often difficult to detect and may lead to devastating medical consequences. For example, the transmission of West Nile Virus (WNV), another flavivirus, from WNV-infected organ donors who were evidently no longer viremic during blood testing to recipients through organ transplantation is well-documented [5,6]. To effectively prevent ZIKV transmission through transfusion of infected blood and particularly, through transplantation of infected organs or tissues, a better understanding of the human cells that could potentially be harboring the infectious virus and serving continuously as a ZIKV reservoir over a prolonged time is needed. The dynamic process of ZIKV clonal selection with specific gene mutations during persistent viral infection and the adaptive mechanisms of human host cells that confer ZIKV persistence will also need to be further explored.

In a study of experimentally ZIKV-infected Rhesus monkeys, ZIKV RNA (v-RNA) was found to persist with the highest levels in hemato-lymphatic tissues, such as lymph nodes and spleen, after they were no longer viremic [7]. Two other studies involving experimentally ZIKV-infected rhesus monkeys also reported that lymphoid tissues and central nervous system (CNS) cells had prolonged viral persistence [8,9]. To investigate possible ZIKV persistence in cells of the hemato-lymphatic system and blood, our laboratory has examined the relative susceptibility of human hematopoietic cell lines with different developmental characteristics to infection by the prototype MR 766 ZIKV strain with a focus on the duration as well as the outcome(s) of the infection [10]. Our study revealed that human B lymphoblastoid cell lines were highly susceptible to ZIKV infection and developed prominent cytopathic changes and fulminant cell necrosis in the culture, while T lymphoblastoid cell lines were very resistant to the ZIKV infection [10]. We reported the human monocytic leukemia/histiocytic lymphoma-originated U937 cell line [11], initially showing a slow rate and only low-grade infection using the ZIKV MR 766 strain, could develop into persistently ZIKV-infected cell lines in which most, if not all, of the cells in the culture were positive for ZIKV antigens despite no apparent cytopathological changes [10]. The ZIKV-infected U937 cell cultures produced ZIKV RNA (v-RNA) and infectious ZIKV persistently (persistent ZIKVs) with distinct infectivity and pathogenicity when tested using various kinds of host cells, in comparison with the original MR766 strain ZIKV [10]. We conducted comparative studies between the original/prototype ZIKVs produced by the acutely infected human U937 cells and the persistently ZIKV-infected human U937 cells, and discussed the genomic variations developed in the persistent ZIKVs [10].

In this study, we similarly examined the relative susceptibility of human hematopoietic cell lines with various developmental characteristics to infection by the prototype ZIKV strains isolated during the contemporary epidemics in America with a focus on the duration as well as the outcome(s) of viral infection. We found that the American isolates of the PRV (Puerto Rico) and FLR (Colombia) ZIKV strains not only failed to infect human T lymphoblastoid cell lines, but also failed to infect human hematopoietic monocytic/histiocytic U937 cells. On the other hand, our study revealed the two American ZIKV strains like the prototype MR 766 ZIKV strain could effectively infect cultured HEK293 human epithelial cell line with documented kidney and neuronal differentiation properties [12].

Consistent with a previous report and the observations that the prototype African ZIKV isolate MR 766 appeared to be more infectious and cytopathogenic to various human cells than the ZIKVs circulating in the Americas [7,11], MR 766 strain ZIKV-infected HEK293 cells completely sloughed off from the flask after developing fulminant cytopathic effects (CPE) and cytolysis in culture. In contrast, small populations of HEK293 cells infected with the PRV and FLR strains of ZIKVs continued to survive the virus-induced cytocidal or cytopathic effects and regrew when they were provided with fresh culture media in the parallel study. Significantly, regrowth of the surviving HEK293 cells in culture resulted in HEK293 cell lines that were persistently infected with the PRV and FLR strains of ZIKV and constitutively producing ZIKV v-RNA genomes and infectious ZIKV virions. We again conducted comparative studies involving genomic variations and alterations of infectivity and cytopathogenicity between persistent ZIKVs produced by the persistently ZIKV-infected human HEK293 cells and the original inoculum or PRV and FLR strains of ZIKV produced by the acutely infected HEK293 cells.

## 2. Results

### 2.1. Infections of Human Epithelial HEK293 Cell Line by Different Strains of ZIKV

HEK293 cells were infected with the MR 766 strain, the PRVABC59 strain (PRV), or the FLR strain of ZIKV at an MOI of 0.1. Three days post infection (POI), cells in all three strains of ZIKV-infected cell cultures showed prominent CPE, rounded up and detached from the culture flasks. The CPE in the infected cell cultures continued to progress. By one-week POI, MR 766 strain ZIKV-infected HEK293 cells essentially lost all the attached cells in the culture. Most of the cells (>60–70%) also sloughed off the flask surface in PRV strain and FLR strain ZIKV-infected cultures. However, occasional islands with a small number of viable cells remained attached on the culture surfaces. The detached dead cells in the two cultures were removed, and the cultures were replenished with fresh culture medium ~10 days after ZIKV infection. Although signs of cytopathic effects and cell degeneration remained prominent in the cultures, evidence of cellular proliferation in the scarce islands could be observed.

### 2.2. Development of Persistently ZIKV-Infected HEK293 Cells

**Figure 1:**
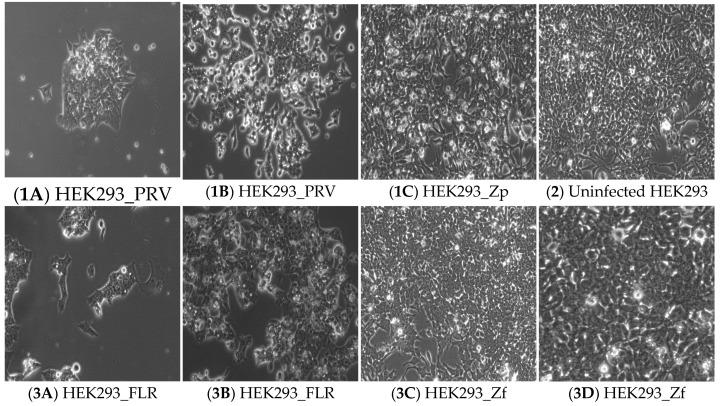
Morphology of HEK293 cells infected by the PRV (Puerto Rico) and FLR (Colombia) strains of ZIKV (ZIKA virus) at different stages. Photo micrographs (100×) of small clusters of viable HEK293 cells continued to adhere on the flasks at week 4 in PRV-ZIKV infected culture (**1A**) and FLR-ZIKV infected culture (**3A**). Proliferation of HEK293 cells was evident at week 6 in PRV-ZIKV infected culture (**1B**) and FLR-ZIKV infected culture (**3B**) after fresh medium was provided. The monolayer HEK293 cells re-established in PRV-ZIKV infected culture (HEK293_Zp, **1C**) and FLR-ZIKV infected culture (HEK293_Zf, **3C**) after fresh medium was provided weekly. Monolayer of non-ZIKV infected HEK293 cells (100×) at Day 5 (**2**). Higher magnification (200×) of the monolayer formed by HEK293_Zf cells (**3D**).

**Figure 2:**
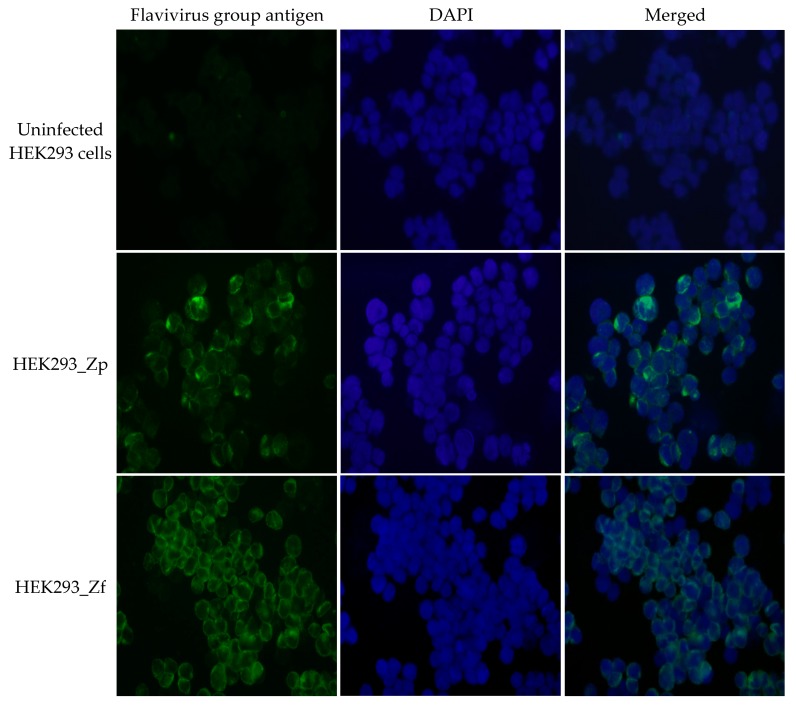
IFA (immunofluorescence assay) of ZIKV-specific antigen in non-ZIKV-infected HEK293 control cells and the monolayer HEK293 cells re-established in PRV strain ZIKV-infected culture (HEK293_Zp) or HEK293 cells re-established in FLR strain ZIKV-infected culture (HEK293_Zf). Cells in the monolayer cultures were trypsinized and dotted onto cover slides before IFA staining. An anti-flavivirus group antigen monoclonal antibody was used as the primary IFA antibody. Merged: the merged image includes the immunofluorescent staining of flavivirus group antigen and DAPI nuclear staining of the cells. IFA images were captured under immunofluorescent microscope at 200×.

**Figure 3:**
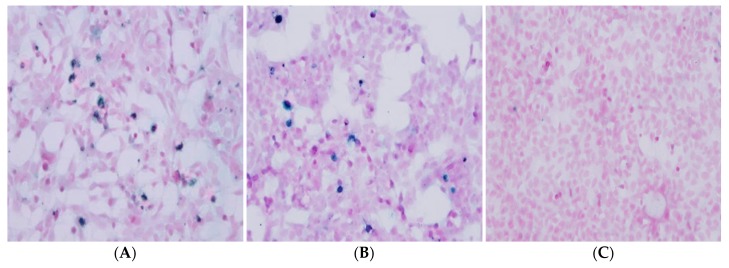
Immunocytochemical staining for cell apoptosis in the monolayer cultures of persistently PRV strain of ZIKV-infected HEK293 cells (HEK293_Zp) and FLR strain of ZIKV-infected HEK293 cells (HEK293_Zf). ApopTag Peroxidase In Situ Apoptosis Detection Kit was used to stain and visualize apoptotic cells. (**A**) HEK293_Zp, (**B**) HEK293_Zf, and (**C**) HEK293 control cell cultures. Images were captured under a research microscope at 200×.

Approximately four weeks after the initial infection, two distinct cell islands with viable cells adhering to the flask in cultures infected with PRV strain ZIKV were observed (Figure 1-1A). Similarly, small clusters of viable cells remained attached to the flask in the cell culture infected with FLR strain ZIKV (Figure 1-3A). The viable cells in the cultures slowly but steadily regrew in the flasks (Figure 1-1B, 1-3B). The cell cultures were first split 6 weeks after infections of ZIKVs. The cells in the 2 ZIKV-infected cultures gradually grew to near confluency (Figure 1-1C, 1-3C). The cultures were then passaged regularly at a 1:5 dilution with fresh culture medium weekly for more than 3–4 months. Cells morphologically resembling the parental HEK293 cells (Figure 1-2), proliferated well in both ZIKV-infected cultures. However, some cells in both cultures continued to exhibit signs of cell degeneration or CPE (Figure 1-1C, 1-3C, 1-3D).

During sub-culturing of the two cell cultures, the trypsinized cells were processed to perform an immunofluorescence assay (IFA) for Zika viral specific antigen using Flavivirus monoclonal antibody 4G2. The IFA showed that the two continuously growing HEK293 cell lines developed after infection with the PRV and FLR strains of ZIKV were persistently infected with the respective ZIKV strains (Figure 2). We designated the two established persistently ZIKV-infected cell lines as HEK293_Zp and HEK293_Zf, for PRV and FLR ZIKV strains, respectively.

### 2.3. Cell Apoptosis in the Cultures of Persistently ZIKV-Infected HEK293 Cell Lines

The newly established persistently ZIKV-infected cell lines, HEK293_Zp and HEK293_Zf, continued to produce degenerating cells and/or cells showing CPE effects. To explore the nature of the degenerating cells found in HEK293_Zp and HEK293_Zf cell cultures, we conducted a TUNEL staining to identify cells undergoing apoptosis [13]. The results presented in Figure 3, show that ~10% and ~5% cells, respectively of the HEK293_Zp and HEK293_Zf cell lines are actively undergoing apoptosis (Figure 3A,B). In comparison, none of the HEK293 cells in the non-ZIKV infected control culture studied in parallel showed evidence of apoptosis (Figure 3C).

### 2.4. Cell Cycle Analysis of Persistently ZIKV-Infected HEK293 Cell Lines

**Figure 4:**
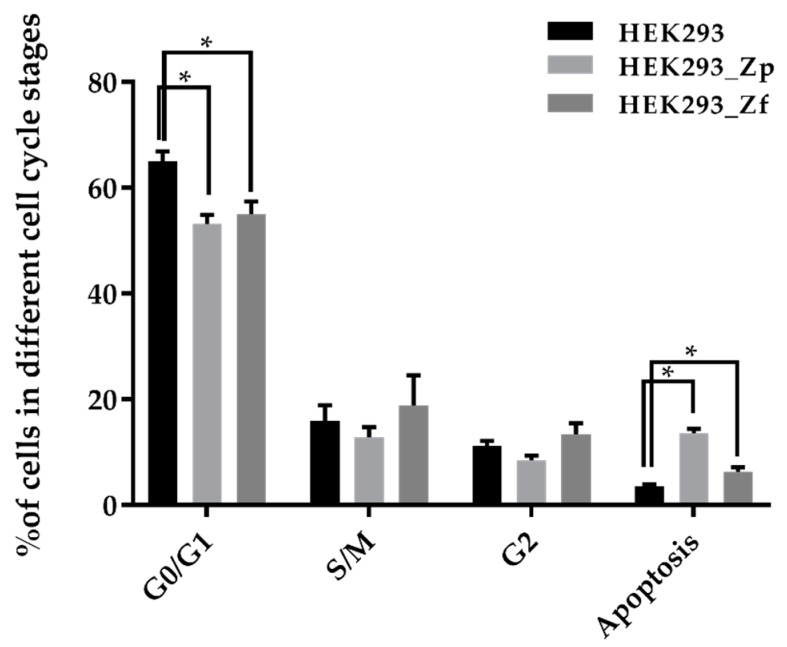
Summary of different cell cycle stages of HEK293 cells with or without persistent PRV and FLR strains of ZIKV infection. The error bars represent the triplicate for each measurement conducted at each time point of the study. Mean ± SD of each condition was calculated and the significance of the difference among and between groups was tested analyzed by ANOVA and student *t*-test. * student *t*-test analysis, *p* < 0.05. G0/G1: Gap 0/Gap 1 phase; S/M: DNA Synthesis/Mitosis phase; G2: Gap 2 phase.

It was reported before that Zika virus infection of human neurospheres alters the cell cycle [14]. We examined the effects of persistent viral infection on the cell cycle and on proliferation of HEK293_Zp, HEK293_Zf, and uninfected HEK293 cells by flow cytometry. In this study, total cell populations of actively growing cell cultures of the two persistently ZIKV-infected HEK293 cell lines along with those in the non-infected control culture, were harvested and the proportion of cells in different cell cycle stages was analyzed. The result of the flow cytometry analysis of the three cell lines is summarized in Figure 4. As expected, the average proportion of apoptotic cells in cell samples of HEK293_Zp and HEK293_Zf cells (13.65% and 6.30%, respectively) were significantly higher than that of non-infected HEK293 cells (3.48%). The average proportions of cells in the S/M and G2 stages were similar among the three cell lines, while the proportions of cells in the G0/G1 stage of HEK293_Zp and HEK293_Zf cells (53.15% and 55.00%, respectively) were significantly lower than that of non-infected HEK293 cells (64.95%). The results also suggest that most of the apoptotic cells present in the HEK293_Zp and HEK293_Zf cell lines might be trapped at G0/G1 stage.

### 2.5. Growth Kinetics and ZIKV Production of the Persistently ZIKV-Infected HEK293 Cell Lines

**Figure 5:**
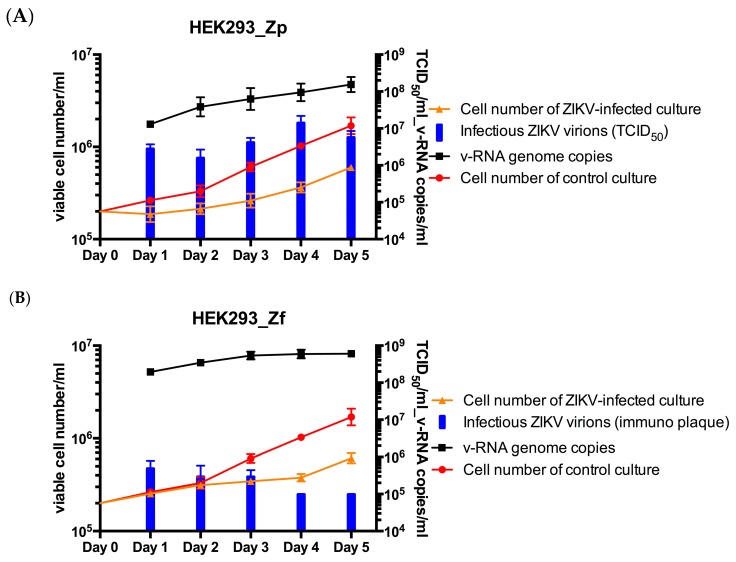
Cell growth, production of ZIKV v-RNA genomes and infectious virions. Panel (**A**) HEK_Zp and panel (**B**) HEK_Zf persistently PRV and FLR strains of ZIKV-infected HEK293 cell cultures. Viable cells were determined by trypan blue dye exclusion. Quantitation of v-RNA ZIKV genomes by RT-PCR and titration of infectious ZIKV virions using the TCID50 assay (immune plaque assay for HEK293_Zf cell cultures) were done using samples collected daily from the supernatants of infected HEK293 cell cultures. The error bars represent the triplicate for each measurement conducted at each time point of the study.

The established HEK293_Zp and HEK293_Zf cell lines were further characterized by studying their cell growth kinetics and ZIKV production abilities. HEK293_Zp and HEK293_Zf cells were seeded in 12-well tissue culture plates at 2 × 10^5^ cells/mL. Culture supernatants were collected daily for five days from three triplicate culture wells of each cell line for titration of infectious ZIKV virions and quantitation of v-RNA genome copies by RT-PCR. After culture supernatant collection, viable cell numbers in each well were determined after trypsinization. Except for a small dip in the HEK293_Zp cell number on the first day, both HEK293_Zp and HEK293_Zf cell lines steadily proliferated at similar rates (Figure 5). However, HEK293_Zp and HEK293_Zf cells with a fraction of the cells continuously undergoing apoptosis in culture, grew at a slower rate compared with non-infected HEK293 control cells. By Day 5, the number of viable HEK293 cells reached about 1 × 10^6^ cells/mL, while cell numbers for both HEK293_Zp and HEK293_Zf only reached about 6 × 10^5^ cells/mL, about half the cell number of the control culture.

HEK293_Zp cells released ~2 × 10^7^ copies/mL of ZIKV genomic RNA in the culture supernatant at Day 1 with a gradual increase to ~2 × 10^8^ copies/mL at Day 5 (Figure 5A). The number of infectious ZIKV virions in culture supernatants titered by the endpoint dilution (TCID 50%) assay against Vero cells also increased gradually from 2 × 10^6^ ID_50_ units/mL at Day 2 to 2 × 10^7^ ID^50^ units/mL at Day 4. The ratio of ZIKV v-RNA genome copies and the infectious virions released into the culture supernatants was ~10.

HEK293_Zf cells released 2 × 10^8^ copies/mL of ZIKV genomic RNA in the culture supernatant at Day 1 and with a gradual increase to 7 × 10^8^ copies/mL at Day 5 (Figure 5B). Despite finding high copy numbers of ZIKV v-RNA genomes in the supernatants of HEK293_Zf cell cultures, it was difficult to titer infectious ZIKV virions by plaque formation with cell necrosis and cytolysis-based endpoint dilution (TCID 50%) assay against Vero cells. It was estimated that ~5 × 10^5^ TCID_50_/_mL_ of infectious ZIKV virions were released into the supernatant of HEK293_Zf cell culture at Day 1 using an immuno plaque assay to count ZIKV antigen-positive infection foci (see below). The number of infectious ZIKV virions in the supernatants did not appear to increase after the first day in culture (Figure 5B). The ratio of ZIKV v-RNA genome copies and the infectious virions released into the culture supernatants was >1000.

### 2.6. Infectivity and Viral Plaque Formation by ZIKVs in Vero Cells

**Figure 6:**
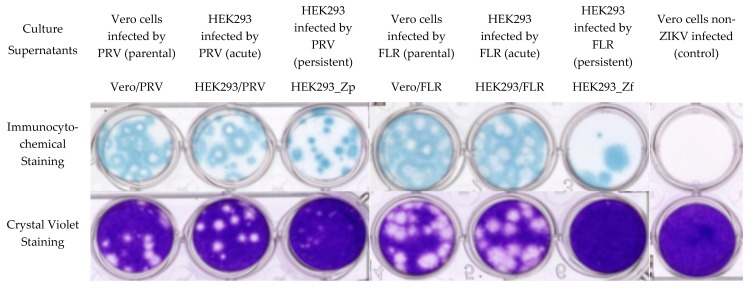
Vero cell cultures infected using ZIKVs collected from the supernatants of acutely and persistently PRV and FLR strains of ZIKV-infected HEK293 cell cultures. Vero control cells as well as Vero cells infected by ZIKVs (PRV_V, PRV_H, Zp_H, FLR_V, FLR_H, Zf_H) using cell culture supernatants (Vero/PRV, HEK293/PRV, HEK293_Zp, Vero/FLR, HEK293/FLR, HEK293_Zf) with the same genomic copy number were stained four days post infection with True Blue after immunoblotting with Anti-Flavivirus Group antigen monoclonal antibody (Clone D1-4G2-4-15, BEI Resources) followed by goat anti-mouse IgG H&L (HRP). Separate sets of culture wells were stained in parallel with Crystal Violet to better visualize plaques formed by the infection of ZIKVs. Immunocytochemical staining showed that the infection foci produced by HEK293_Zp-generated persistent ZIKV (Zp_H) had poorly-formed lytic centers. HEK293_Zf-generated persistent ZIKV (Zf_H) produced significantly fewer infection foci. Moreover, the infection foci formed by Zf_H had no lytic centers.

We compared the cytopathogenicity of ZIKVs produced by persistently ZIKV-infected HEK293 cells and those of the original/parental strains of ZIKVs by examining their infectivity and viral plaque formation ability on monolayer culture of Vero cells. Vero cells seeded in a 24-well tissue culture plates were inoculated with culture supernatants containing the same viral genome copies from (1) Vero cell cultures infected by the parental PRV and FLR strains of ZIKV, (2) HEK293 cell cultures acutely infected (four days post infection) by the parental PRV and FLR strains of ZIKV and (3) persistently ZIKV-infected HEK293_Zp and HEK293_Zf cultures. The foci of ZIKV infection in the fixed culture plates of Vero cells were analyzed by immunochemical staining against ZIKV antigen four days after viral infections. Plaque formations associated with cytopathogenicity and cytolysis following viral infections were counted in the culture plates after True Blue staining (Figure 6).

The PRV strain of ZIKV propagated both in Vero cells (PRV_V) and in acutely infected HEK293 cells (PRV_H) effectively infected Vero cells in the culture plates and produced infectious foci as well as plaques with similar morphology and size. The FLR strain of ZIKV propagated in both the cultures of Vero cells (FLR_V) and acutely infected HEK293 cells (FLR_H) similarly infected Vero cells effectively in the culture plates and produced infectious foci as well as plaques with similar morphology and size. However, it is important to note that the size of plaques produced by FLR strain ZIKVs in the monolayer culture plates of Vero cells were noticeably larger than those produced by PRV strain ZIKVs (Figure 6). The persistent ZIKV, Zp_H, produced by the persistently PRV strain ZIKV-infected HEK293 cells could also infect Vero cells efficiently, but produced only very small plaques on the monolayer culture plates of Vero cells four days after infection. The persistent ZIKV, Zf_H, produced by the persistently FLR strain ZIKV-infected HEK293 cells could infect Vero cells with low efficiency, but failed to induce distinguishable plaque formation with clear cytolysis (Figure 6).

### 2.7. Comparative Genomic Analysis of ZIKVs Produced by Acutely and Persistently ZIKV-Infected HEK293 Cells

We next compared the genomes of two persistent ZIKVs, Zp_H and Zf_H, produced by the two persistently ZIKV-infected HEK293 cell lines (HEK293_Zp and HEK293_Zf, respectively), with those of their parental viruses PRV_V and FLR_V (inoculum), produced originally by Vero cells. We also included in the comparison of ZIKVs PRV_H and FLR_H produced by HEK293 cells acutely infected (four days post infection) by the parental PRV_V and FLR_V strains of ZIKV. Viral genomic RNAs were extracted from virions in the culture supernatants harvested by PEG-precipitation. The RNAs were processed for v-RNA genomic sequencing using the MiSeq platform. The qualified raw reads were assembled based on the previously reported reference genome sequences of ZIKV PRV (GenBank Accession number: KX601168) and ZIKV FLR (GenBank Accession number: KX087102), respectively. The nucleotide positions of the ZIKV reference genome sequence was used as the coordinate in our comparative ZIKV genomic study. Nucleotide variations or mutations with frequency less than 10% were excluded from the comparison. Table 1 and Table 2 summarize the results of variant calling showing the positions and types of nucleotide changes and the corresponding amino acid changes.

The comparative genomic sequences of various isolates of ZIKV PRV are listed in Table 1. The inoculum (BEI-derived) virus PRV_V produced originally by Vero cells and the ZIKV PRV_H produced by HEK293 cells acutely infected (four days post infection) by the inoculum PRV_V strain of ZIKV had sequence heterogeneities at nucleotide positions 1947, 2763 and 5662, compared to the reference PRV_V strain ZIKV genome sequence (GenBank Accession number: KX601168). The genomic sequences of the three persistent ZIKVs of PRV strain, Zp_H, produced by the persistently ZIKV-infected HEK293 cell line HEK293_Zp 8–10 months post infection showed more nucleotide heterogeneity at many different positions in various NS genes (highlighted in Table 1). However, no consistent sequence changes were identified in the ZIKV envelope and membrane protein genes. No compensatory changes in the genomic RNAs were observed.

The results of the genomic analysis of the three persistent ZIKVs, Zf_H, produced by persistently ZIKV-infected HEK293 cell line (HEK293_Zf) 8–11 months post infection, in comparison with the inoculum virus FLR_V propagated by Vero cells as well as ZIKV FLR_H produced by HEK293 cells acutely infected (four days post infection) by the inoculum FLR_V strain of ZIKV are present in Table 2. The genomic sequence of FLR_V was found to be identical to the reported reference FLR strain ZIKV genome sequence (GenBank Accession number: KX087102). The genomic sequence of ZIKV FLR_V produced by HEK293 cells acutely infected (Day 4) by the inoculum FLR_V strain of ZIKV showed sequence heterogeneity at positions 1522, 3878 and 8794. The genomic sequences of the three persistent ZIKVs of FLR strain, Zf_H, produced by the persistently ZIKV-infected HEK293 cell line (HEK293_Zf) 8–10 months post infection showed more nucleotide variations or heterogeneity in many different positions of various NS genes as well as the envelope gene. The variations or mutations occurred at nucleotide positions 3338, 3878, 6503, 7156 and 8140 (highlighted in Table 2) in various NS genes appeared to be the most consistent and stable. In addition, the mutation at nucleotide position 715 of precursor protein gene also appeared to be rather stable. The stable mutations in these genes in the genome of persistent ZIKV, Zf_H, could be related to its attenuated properties in cell infectivity and cytopathogenicity in Vero cells and various human epithelial cells.

## 3. Discussion

The recent ZIKA virus epidemic is associated with fetal brain lesions and other serious birth defects (including microcephaly, fetal growth restriction, stillbirth, ocular disorders, and CNS injury, etc.) classified as congenital ZIKV syndrome. Postnatal ZIKV infection in infants and children has been reported, and one study has shown that postnatal ZIKV infection is associated with persistent abnormalities in brain structure, function, and behavior in infant macaques [15]. However, there is no scientific documentation of ZIKV-associated birth defects in Africa, where the virus historically originated. The study by Zhang et al. claimed that microcephalies caused by American isolates/strains of ZIKV in neonatal mice are more severe compared to that caused by an old Asian strain of ZIKV [16].

Previous studies conducted by our laboratory showed that the prototype MR 766 strain ZIKV initially developed only a low-grade infection at a slow rate in the human monocytic/histiocytic U937 cell line without causing CPE changes or cytolysis could lead to ZIKV persistency. The continuously growing persistently ZIKV-infected U937 cell lines were constantly producing ZIKV v-RNA genomes and infectious virions with alterations of infectivity and cytopathogenicity [10]. Our current study revealed the American isolates including the PRV and FLR ZIKV strains failed to infect the human hematopoietic U937 cell line. However, the American PRV and FLR ZIKV isolates could effectively infect human epithelial HEK293 cell lines derived from human embryonic kidney cells known of exhibiting many properties of immature neurons [12]. Infections by the PRV and FLR ZIKV strains quickly resulted in prominent CPE changes and extensive cytolysis in cultures of HEK293 cells. Interestingly, persistently ZIKV-infected HEK293 cell lines could also be established from PRV and FLR strains of ZIKV-infected cultures by providing the few surviving cells in the ZIKV-infected cultures with fresh media. The persistently ZIKV-infected HEK293 cell lines also constantly produced ZIKV v-RNA genome and infectious virions in culture. Different from the persistently MR 766 strain ZIKV-infected U937 cells previously established, some of the cells in the continuously growing PRV and FLR strains of ZIKV-infected HEK293 cell cultures would evidently continue to undergo apoptosis.

**Figure A1:**
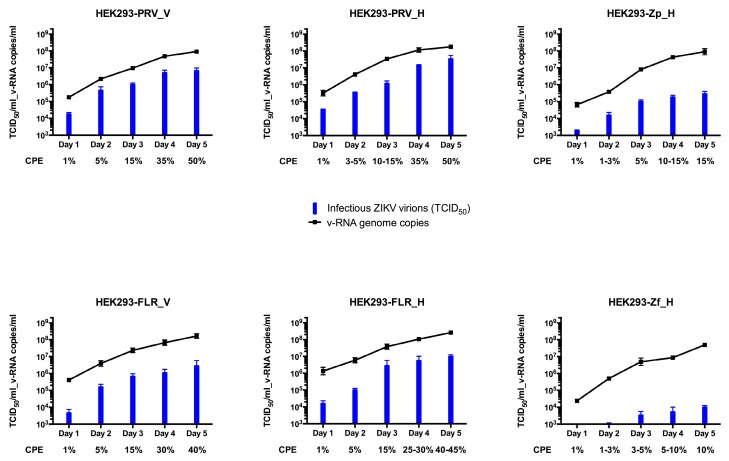
Production of ZIKV RNA genomes and infectious virions in naïve HEK293 cells infected with ZIKVs present in the supernatants of acutely and persistently PRV and FLR strains of ZIKV-infected cultures. HEK293 cells were infected by ZIKVs (PRV_V, PRV_H, Zp_H, FLR_V, FLR_H, Zf_H) using supernatant aliquots with the same genomic copy number from 6 cell cultures (Vero/PRV, HEK293/PRV, HEK293_Zp, Vero/FLR, HEK293/FLR, HEK293_Zf). Quantitation of v-RNA ZIKV genomes by RT-PCR and titration of infectious ZIKV virions by the TCID50 assay were examined using supernatants of cell culture aliquots collected daily. The error bars represent the triplicate for each measurement conducted at each time point of the study.

The findings of only a small population of PRV and FRL strains ZIKV-infected HEK293 cells could survive and upon favorable conditions, would gradually propagate to confluency in culture over time were reproduced multiple times. The growing HEK293 cells that survived infections of these ZIKV strains in the culture were found persistently infected by the virus with positive IFA for Zika viral specific antigen. A similar finding was reported previously in the other flavivirus-infected cultures of HEK293 cells and Vero cells [17,18]. The present study was conducted comparatively on persistent ZIKVs produced from a set of the persistently American strains ZIKV-infected HEK293 cell lines established in culture and then followed closely for more than 11 months. We compared the infectivity and cytopathogenicity of the persistent ZIKVs produced by persistently ZIKV-infected HEK293 cells with the parental/original ZIKVs produced by the acutely ZIKV-infected HEK293 cells to Vero cells (Figure 5), naïve HEK293 cells (Figure A1) and various human epithelial cell lines (HeLa cell line and A549 cell line, data not shown). The persistent PRV and FLR ZIKV strains had evidently attenuated cell infectivity and cytopathogenicity compared to the original inoculum PRV and FLR ZIKV strains of ZIKVs. Our previous study showed that the persistent ZIKVs produced by persistently MR766 strain ZIKV-infected human U937 cells established in three independent studies had properties not only distinct from the original inoculum ZIKV, but also among themselves in terms of specificity of cell infection and cytopathogenicity [10]. Importantly, in contrast to the finding in the present study, all the persistent ZIKVs produced by the persistently MR 766 strain ZIKV-infected U937 cell lines developed in the three studies of 1st round of ZIKV persistence were found highly infectious and cytopathogenic to the naïve U937 cells. Genomic analyses of ZIKVs showed that in comparison to the original MR 766 ZIKV strain, the persistent MR 766 strains were three rather uniform subclones of ZIKVs, each with distinct sets of mutations in various key genes enabling the selected viruses to continuously infect and proliferate in U937 cells with apparently activated anti-viral mechanisms [10].

Establishment of persistence in ZIKV-infected HEK293 cells resembles more closely to the establishment of the second round MR766 strain ZIKV persistence, which was achieved by infecting the naïve U937 cells with the three persistent ZIKVs obtained in the first round of ZIKV persistence in U937 cells. Differing from a low rate and low-grade infection by the original/parental MR766 strain ZIKV in U937 cells, large numbers of naïve U937 cells in the cultures infected by these persistent ZIKVs produced by U937 cells in the first round of ZIKV persistence quickly became IFA-positive for ZIKV antigen, developed prominent CPE changes and underwent cytolytic necrosis. Only a small number of cells could continue to survive and grow in the ZIKV-infected U937 cell culture. In similar instance, the PRV and FLR strains of ZIKV are highly infectious and cause cytopathic effects in HEK293 cells. Only the few of the HEK293 cells that could quickly adapt metabolically to accommodate viral persistence following ZIKV infection could continue to survive in the ZIKV-infected cultures. Therefore, the process of both developing the second round MR 766 strain of ZIKV persistence in U937 cells and establishing persistently PRV and FLR strains ZIKV-infected HEK293 cells appeared to mainly involve selection of the very few members of human blood or epithelial cells with adaptive metabolic functions enabling the cells to continuously survive and grow without actively undergoing apoptosis or cytolysis, despite active intra-cellular propagation of ZIKVs. The comparative genomic study of the persistent ZIKVs produced by the persistently MR 766 strain ZIKV-infected U937 cells established in the first round of ZIKV persistence without having detectable CPE and cytopathogenicity and the second round of ZIKV persistence with prominent CPE and cytopathogenicity revealed positive or negative of stable nucleotide variations or mutations in the key genes compared to the respective inoculum ZIKVs [10].

The comparative genome sequence analyses of persistent ZIKVs produced by the persistently PRV and FLR ZIKV strains ZIKV-infected HEK293 cells and the original inoculum of ZIKV or ZIKVs produced during the acute infection of HEK293 cells showed that the genomes of both persistent PRV and FLR ZIKVs were genetically more diverse and heterogenous with more nucleotide variations in different nucleotide positions of different genes (Table 1 and Table 2). In the prolonged process of establishing persistently ZIKV-infected cultures, the populations of ZIKVs would gradually become more heterogenous without a definite selection pressure on the infecting inoculum viruses. Importantly, our genomic analyses revealed that there were apparently different processes of viral selection in the intricate interplays of between infecting PRV and FLR ZIKV strains with the HEK293 host cells. The genomes of persistent ZIKVs, Zp_H, produced by the persistently PRV strain ZIKV-infected HEK293 cell line developed after an initial fulminant cytopathic ZIKV infection did not show any consistent nucleotide changes or stable variations. In this context, a recent study of ZIKV genomes in the persistently ZIKV-infected culture of human fetal astrocytes also reported no high degree of stable variation found [19]. In contrast, the genomes of persistent ZIKVs, Zf_H, produced by the persistently FLR strain ZIKV-infected HEK293 cell line developed after highly cytopathic ZIKV infection had stable variations at several positions in various NS genes and one in precursor protein gene (Red shallow in Table 2). Specific clones of FLR strain ZIKV with the mutations or stable variations in these genes were evidently selected in the process of developing viral persistence in the ZIKV-infected HEK293 cells. Further studies are needed to elucidate how these nucleotide variations could affect the cell infectivity and cytopathogenicity of the persistent ZIKV, Zf_H, on Vero cells and other human epithelial cells. The findings of fixed patterns of mutations or at times lack of fixed patterns of mutations in genomes of the 2 persistent ZIKVs produced by the persistently FLR and PRV strains ZIKV-infected HEK293 cells showed there is more than one way to establish viral persistence in the ZIKV-infected human host cells.

The present study and our previous study [10] revealed that both low grade with slow rate ZIKV infections that produced no detectable CPE and high grade with rapid rate ZIKV infections that produced prominent CPE as well as extensive cell necrosis could lead to ZIKV persistence. However, the mechanisms of developing viral persistence for the ZIKVs in infected human cells could be very different. Both viral strains/isolates and cellular mechanisms of infected host cells play an important role in the development of ZIKV persistence in human cells of different developmental characteristics. The processes and the outcomes of establishing ZIKV persistence are highly complex. Our studies revealed that infections by ZIKVs with low infectivity and cytopathogenicity could lead to selection of more virulent ZIKVs, some with even altered cell infection specificity, produced by the persistently ZIKV-infected cells. On the other hand, infections by ZIKV with high infectivity and cytopathogenicity in human epithelial cells may also lead to selection of less virulent viruses produced by the persistently ZIKV-infected cells.

The possible clinical relevance of the study results includes the apparent new challenge in detection of the persistent ZIKVs with genomic sequence variations in the persistently ZIKV-infected cells in tissues. In addition, the finding that infections of HEK293 cells with neuronal differentiation properties by the two American isolates of ZIKV, but not the prototype ZIKV MR766 strain, could result in viral persistence in the cells may also have its clinical relevance [12]. The two seemingly less pathogenic American isolates of ZIKV that can establish persistence in the human cells with neuronal developmental characteristics may be playing a previously unappreciated role in the recent ZIKA virus epidemic associated with fetal microcephaly of congenital ZIKV syndrome [12]. Our on-going comparative gene expression study for neuronal differentiation and development in HEK293 cells with or without persistent ZIKV infections may shed some lights on the relevance of ZIKV-related neurological abnormalities in recent ZIKV epidemic. In this context, it is also unclear how PRV and FLR isolates with similar genetic background showed two dissimilar patterns of genomic changes during the persistent infection. Further study will also be needed to verify if the dissimilar patterns found are indeed strain-specific. Evidently, the cell culture study will likely be over simplistic in comparison with the complexity of various clinical conditions. However, the cell culture study could yield important and useful information that may not be easily attenable clinically. We anticipate that the study will further contribute to the understanding of the fundamental biology of adaptive mutations and selection for both the infecting viruses and the infected human host cells during development of ZIKV persistence. The persistently ZIKV-infected human cell lines that we have developed should also be useful for investigating critical molecular pathways of ZIKV persistence and to study drugs or countermeasures against ZIKV infections and transmission.

## 4. Materials and Methods

### 4.1. Virus and Cells

Zika virus stocks, PRVABC59 strain (Puerto Rico strain, NR-50244) and FLR strain (Colombia strain, NR-50241) were obtained from BEI Resources, NIAID/NIH (ATCC, Manassas, VA, USA). Cell lines used in this study include: human embryonic kidney cells, HEK293 (ATCC CRL-1573), African green monkey kidney cells, Vero (ATCC CCL-81). Cell lines were purchased from ATCC (Manassas, VA, USA). Vero cells were maintained in RPMI 1640 medium containing L-glutamine and 25 mM HEPES (Corning, Corning, NY, USA) supplemented with 10% fetal bovine serum (FBS) and 1% penicillin-streptomycin solution (P/S) at 37 °C with 5% CO_2_; all the other cell lines were grown in Minimum Essential Medium (DMEM, Corning) supplemented with 10% FBS and 1% P/S solution.

### 4.2. Propagation of the ZIKV PRV and ZIKV FLR Working Stocks

The working Zika virus stocks were prepared as we reported previously [10]. Vero cells were cultured in a T-75 culture flask to 90% confluent. After aspirating medium from the cell culture, Vero cells were inoculated with ZIKV suspension in 2 mL of RPMI 1640 medium containing 2% FBS and 1% P/S (R2 medium). Viral inoculums were prepared from ZIKV stocks obtained from BEI Resources at multiplicity of infection (MOI) of 0.1 ± 0.01 according to the package description. The infected Vero cell cultures were incubated at 37 °C for 1 h with gentle rocking very 15 min. After viral absorption, 10 mL of R2 medium was added to the cell culture, the ZIKV-infected Vero cell cultures were incubated in a CO_2_ incubator at 37 °C and monitored daily for the appearance of viral CPE. When ~80% of cells detached from the culture surface of the flask after developing CPE, the culture supernatants were harvested by centrifuging for 10 min at 1000× *g*. The culture supernatants were passed through a 0.22 µm filter to remove cell debris, aliquoted, tittered and stored at −80 °C as the ZIKV working stocks to be used in the subsequent experiments.

### 4.3. Infection of HEK293 Cell Line with Zika Virus

HEK293 cells were seeded in a T-75 tissue culture flask, cells grew to full confluence, which contained approximately 2–3 × 10^7^ cells per flask. To infect HEK293 cells, 3 mL of R2 medium containing ~3 × 10^6^ p.f.u. of ZIKV PRV or ZIKV FLR working stock was added to each flask, at MOI of about 0.1, and incubated at 37 °C for 2 h with frequent rocking. After incubation, cells were washed with PBS, and 30 mL of DMEM culture medium supplemented with 10% FBS was added to the cell culture. The infected cell cultures were maintained at 37 °C with 5% CO_2_. Cell cultures were monitored daily to observe any cell morphology changes and CPE. To harvest acute-infected virus produced in HEK293 cells, culture supernatants were collected by centrifugation 4–5 days after infection. Culture supernatants were stored at −80 °C for viral titration and viral genome copy estimation.

To establish persistently ZIKV-infected cell cultures, fresh culture medium was replenished to the ZIKV-infected HEK293 cell cultures after degenerated cells detached from culture surface were removed. The cell cultures were continuously maintained until surviving cells re-populated the cultures and the cultures could be passed continuously.

### 4.4. Immunofluorescence Assay (IFA)

To prepare cell samples for immunofluorescence assay (IFA), cell culture was trypsinized, cell suspension was centrifuged (1000× *g*, 10 min), and cell pellet was washed once with phosphate buffered saline (PBS). After centrifugation, the washed cell pellet was re-suspended in 10–20 µL of PBS and cell suspension was dotted on a microscopic slide. The slides were air-dried and then fixed with a mixture of methanol: acetone (1:1) for 5–10 min at room temperature. The procedure for IFA detection of ZIKV-specific antigen has been previously described [10]. Anti-Flavivirus Group antigen monoclonal antibody (Clone D1-4G2-4-15, BEI Resources) was used as the primary antibody for IFA to detect cells that were positive for producing ZIKV antigen, in order to detect the presence of ZIKV antigenic proteins in cells. Cell dots were blocked for 30–60 min with a blocking solution (KPL, Gaithersburg, MD, USA), and 1% bovine serum albumin (BSA) in PBS containing 100 µg/mL of human immunoglobulin. After removing the remaining liquid, cell dots were then incubated with primary antibody (1:300 dilutions in 1% BSA-PBS) at room temperature for 30–60 min in a humidified chamber. After washing with PBS 3 times, the cell smears were incubated with a secondary antibody, Alexa Fluor 488 conjugated goat-anti-mouse IgG (1:400) (Jackson ImmunoResearch Labs, West Grove, PA, USA), for 15–20 min. The slide was again washed with PBS 3 times and then once with distilled water. The slides were air dried and mounted with glycerol-PBS mixture containing DAPI (4′,6-diamidino-2-phenylindole, Sigma, St. Louis, Mo, USA). The cell images were captured using an up-right immunofluorescent microscope with attached digital camera system (Olympus BX51, Olympus, Center Valley, PA, USA).

### 4.5. Assay of Apoptosis

Apoptosis of cells was detected using TUNEL (TdT-mediated dUTP-biotin nick end-labeling) method [13]. Cell cultures were seeded on a circular cover-glass in a 12-well tissue culture plate and cultured for 3–4 days. Cell slides were washed twice with PBS and fixed with 3.7% formaldehyde in PBS for 2 h at room temperature. Fixed cells were washed with PBS and permeabilized with 0.1% Triton X-100 PBS containing 1% BSA and 50 mM glycine for 30 min at room temperature. To stain the apoptotic cells, an ApopTag Peroxidase In Situ Apoptosis Detection Kit (MilliPore, Burlington, MA, USA) was used following the manufacturer’s suggested protocols. Cell slides were incubated with a True Blue peroxidase substrate solution (KPL) to visualize the apoptotic cells. The stained cell slides were imaged using a research microscope with digital camera system (Olympus BX51, Olympus).

### 4.6. Cell Cycle Analysis

To perform cell cycle analysis, HEK293 cells (infected or non-infected) were seeded in T-25 cell culture flasks at 1–2 × 10^6^ cells per flask and incubated at 37 °C for 3 days. The total cells in each flask were harvested by trypsinization, cell pellets were washed with PBS and then fixed with cold (−20 °C) 70% ethanol by adding ethanol drop by drop into each cell pellet while gently vortexing. The fixed cell suspensions were stored in a −20 °C freezer. To conduct cell cycle analysis, the fixed cell pellets were washed first with PBS, and then with staining buffer (2% FBS, 0.1% NaN_3_ in 1 × PBS) by centrifuging for 10 min at 1500 ×g and aspirating the supernatant. Approximately 1 × 10^6^ cells were re-suspended in 0.1 mL of staining buffer, 20 μL 7-AAD (BD, Franklin Lakes, NJ, USA) was added into each tube and incubated for 15 min in the dark at room temperature. Cell cycle analysis was performed using a flow cytometer (BD LSRFortessa X-20, BD). Assay was performed in triplicate.

### 4.7. Viral Production Kinetics of Persistently ZIKV-Infected HEK293 Cells

To study ZIKV production kinetics of persistently infected HEK293 cells, infected cells were seeded in 12-well tissue culture plates at 2 × 10^5^ cells/well in 1 mL of culture medium and incubated at 37 °C with 5% CO_2_. Culture supernatants (0.4 mL) in 3 culture wells of each cell line were collected daily for viral titration and viral genome copy estimation. After supernatant collection, total cells in each well were harvested by trypsinization, and viable cells in each culture well were enumerated using a hemocytometer. Triplicate wells were used for each experiment. The experiment was performed for 5 days.

### 4.8. Endpoint Dilution Assay (TCID50)

The ZIKV infectious titer of culture supernatant was estimated as the 50% tissue culture infective dose (TCID_50_), using Vero cells as the assay target. The procedure has been reported in our previous study [10]. Exponentially growing Vero cells were seeded in 96-well culture plates at 2 × 10^4^ cells/well in RPMI 1640 medium containing 10% FBS one day before performing the assay. Aliquots of cell culture supernatants to be titrated were 10-fold serially diluted in R2 medium. To perform the assay, the culture medium of the prepared 96-well plates of Vero cells was aspirated and 20 µL of serially diluted culture supernatant to be tested was rapidly added into each well. A total of 8 wells were inoculated for each serial dilution. After incubating the plates at 37 °C for 2 h for viral absorption with frequent rocking every 15–30 min, 150 µL of fresh R2 medium was added into each well. The plates were incubated in a CO_2_ incubator at 37 °C and monitored for ~5 days. At the end of the assay, the numbers of culture wells for each dilution point showing clear viral CPE were recorded. The TCID_50_ titer was calculated based on the method described by Hierholzer and Kilington in the first edition of the Virology Methods Manual. Assay was conducted in triplicate.

### 4.9. Real-Time One-Step Reverse Transcription-Quantitative PCR (RT-qPCR)

Quantification of v-RNA of ZIKV genome was performed by a real-time RT-PCR assay using a ZIKV-specific primer set suitable for detecting both PRV and FLR ZIKV strains. The assay procedure has been previously reported [10]. Total RNA of each culture supernatant was extracted from 140 µL of sample stored at −80 °C using QIAamp Viral RNA Mini Kit (Qiagen, Dusseldorf, Germany), following the manufacturer’s protocol. The v-RNA copy numbers in purified RNA were quantified using iTaq Universal SYBR Green One-Step Kit (Bio-Rad, Hercules, CA, USA) and Bio-Rad CFX96 system (Bio-Rad). Each reaction mixture of one-step RT-qPCR contained 5 µL of purified RNA, 10 µL of iTaq universal SYBR Green reaction mix, 0.25 µL of iScript reverse transcriptase, 0.5 µL each of 10 µM forward and reverse primers, and 4.25 µL of nuclease-free water, to a final volume of 20 µL. The RT-qPCR was started from 50 °C reverse transcription step for 30 min and followed by PCR amplification with 95 °C pre-heat for 1 min and then 45 two-step thermocycles at 95 °C for 10 s and 57 °C for 30 s. The ZIKV-specific primer pair used in the RT-qPCR reaction was described previously [20]. The sequences of the forward and reverse primers are: 5′-CCTTGGATTCTTGAACGAGGA-3′ and 5′-AGAGCTTCATTCTCCAGATCAA-3′. The standard curve for estimating v-RNA copy number present in the tested sample was calculated by fitting the Ct value to a strand curve. The standard curve was generated by RT-qPCR run against 5 RNA samples prepared from a 10-fold serial dilution of the ZIKV PRV genomic RNA solution containing 2.0 × 10^4^ ZIKV RNA genome copies/µl (or an RNA solution containing 4.4 × 10^4^ ZIKV RNA genome copies/µL for ZIKV FLR) provided by BEI. Quantification was performed in triplicate.

### 4.10. Plaque Assay

Approximately 5 × 10^4^ Vero cells were seeded into 24-well plates 24 h before the experiment within 1 mL of RPMI 1640 medium containing 10% FBS. Supernatants from ZIKV-infected cell cultures were diluted with R2 medium to achieve equal copy number of ZIKV v-RNA in the same volume. The culture medium of the 24-well plates of Vero cells was aspirated and 200 µL of the prepared culture supernatants were added into each well (in duplicate). After incubating the plates at 37 °C for 2 h for viral absorption (with gentle rocking every 15 min), the infected cells were washed with PBS after aspirating the inoculums from the wells, 1 mL overlay medium was added into each well. The plates were incubated in a CO_2_ incubator at 37 °C and monitored for 3–5 days. The overlay medium was prepared by adding 0.5% agarose to RMPI 1640 medium supplemented with 2% FBS and antibiotic solution. After incubation, cells were fixed with 2 mL/well of 3.7% paraformaldehyde in PBS for 2–4 h. The agarose overlay and formaldehyde solution were removed from culture wells. Cells were rinsed with water and stained with either 0.5% crystal violet in 50% ethanol or True Blue (KPL) after immunoblotting with Anti-Flavivirus Group antigen monoclonal antibody (Clone D1-4G2-4-15, BEI Resources) followed by goat anti-mouse IgG H&L (HRP) (ab6789, Abcam, Cambridge, MA, USA).

The images of stained cell culture plates were recorded using a GE imaging system (Amersham Imager 6000, GE, Pittsburgh, PA, USA).

### 4.11. Infectivity of Zika Virus Produced from Persistently Infected Cells on Vero Cell Lines

Vero cells were seeded in 12-well plates at 1 × 10^5^ cells/well in 1 mL of culture medium one day prior to conducting the experiment. At Day 0, culture medium in each culture well was aspirated, and 400 µL of various ZIKV suspensions in R2 medium was added to each well at an MOI at 0.1. After incubating the plates at 37 °C for 2 h for viral absorption (with frequent rocking), viral inoculums in each culture well were aspirated. The infected cells were rinsed with PBS, and 2 mL of fresh culture medium was added into each well. The plates were incubated in a CO_2_ incubator at 37 °C. Cell cultures were monitored for morphological changes daily, and 0.5 mL of culture supernatant from each well was collected for viral titration and estimation of viral genome copies. The culture plates were replenished daily with 0.5 mL of fresh culture medium after sample collection to maintain the culture volume of each well at 2 mL. A total of 5 sample of culture supernatants were collected for each well and stored at −80 °C.

### 4.12. Viral RNA Preparation for Viral Genome Sequencing

To prepare viral RNA for genome sequencing, we followed a previously described procedure [10]. RNAs of viral particles were extracted from the cell culture supernatant. Twenty mL of culture supernatant from acutely or persistently infected cells was harvested. Each supernatant was centrifuged at 2000× *g* for 10 min to remove cell debris and passed through a 0.22 µm filter to further remove cellular residuals. The supernatant was chilled on ice and 10 mL of 24% PEG (polyethylene glycol)-8000 solution (with 2.5M NaCl) was added to the culture supernatant. The PEG-supernatant mixture was rocked at 4 °C, overnight. The mixture was then centrifuged at 3000× *g* at 4 °C for 30 min. The precipitate was re-suspended in 100 µL of PBS after the supernatant was discarded. Trizol Reagent, 300 µL, was added to the viral suspension to extract viral RNA using Direct-zol MiniPrep kit (Zymo Research, Irvine, CA, USA), following the procedures provided by the manufacturer. The extracted RNA samples were quality checked and quantified by RNA pico chips of 2100 Bioanalyzer (Agilent, Santa Clara, CA, USA).

### 4.13. RNA-Seq of Viral Genome

RNA-Seq of viral genomes was studied using an Illumina Miseq platform following the previously reported procedure [10]. The viral RNAs from various culture supernatants were used to generate libraries using an Illumina TruSeq stranded total RNA sample prep kit, following the manufacturer’s standard procedures. In brief, total RNA was fragmented in the presence of divalent ions at 94 °C for 8 min. Fragmented RNAs were reverse-transcribed into first-strand cDNAs, and then second-strand cDNAs. The double strand cDNAs were adenylated at the 3′-ends, and then ligated with sequencing index adaptors. After amplifying for 15 cycles. The cDNA Libraries were loaded in one MiSeq Nano Flow Cell using MiSeq Reagent Nano kit for a 100-cycle paired-end sequencing on the MiSeq sequencer (Illumina, San Diego, CA, USA).

### 4.14. Analysis of the Viral Genome Variants

To study viral genomes and variation, a previously reported procedure was followed [10]. The fastq files generated from the Illumina MiSeq sequencer were used for viral genome variant analysis. All the sequence analyses were performed using the software package of CLC Genomics Workbench version 11.0.1. The raw reads were trimmed to remove the regions with more than two ambiguous bases and regions having 5% of the bases with quality score lower than 20. The trimmed raw reads were then mapped to the reference sequence of the ZIKV PRVABC59 (Puerto Rico strain) (GenBank Accession number: KX601168) or the ZIKV FLR (Colombia strain) (GenBank Accession number: KX087102) with the mapping parameter: match score = 1, Mismatch cost = 2, insertion cost = 3, deletion cost = 3, mapped length fraction > 0.5, and similarity fraction > 0.8. The local realignment was applied to realign the unaligned ends from the reads mapping. The aligned reads tracked from the local realignment were subject to Low Frequency Variant Detection workflow with the parameter: Ploidy = 1, required variant probability = 80%, the minimum coverage = 5, minimum reads count = 2, minimum frequency = 1%.

### 4.15. Statistical Analysis

All continuous variables are presented as mean ± SD. Data among groups were analyzed using ANOVA followed by student-t test. Values of *p* < 0.05 were considered statistically significant.

## Figures and Tables

**Table 1:** Summary of ZIKV RNA genomic variants bearing sequence changes in structural proteins identified in the inoculum PRV ZIKV and ZIKVs produced by acutely and persistently PRV ZIKV-infected HEK293 cells on the structural protein genes in comparison with the reported PRV reference genomic sequence.

Range on Reference Sequence	Encoded Protein	Type	nt Position	Reference Base	Variant	Amino Acid Change	Allele Frequency				
							PRV_V (Inoculum)	PRV_H (4d pi)	Zp_H (8mo pi)	Zp_H (9mo pi)	Zp_H (10mo pi)
	5’UTR	Del	40	A	-				18.8		
456–734	Protein pr	SNV	516	G	A	Glu143Lys				28.0	
		SNV	714	G	A	Glu209Lys			37.0	82.1	
735–959	Membrane glycoprotein M	SNV	843	T	C	Phe252Leu					12.6
960–2471	Envelope protein E	SNV	1001	T	C	-				32.0	
		SNV	1244	G	T	-				34.4	
		SNV	1531	A	C	Glu481Ala				11.4	
		SNV	1937	C	T	-			10.9		
		SNV	1947	T	G	Leu620Val	20.9	13.0			
		SNV	2485	C	T	Ser799Leu				10.7	
		SNV	2670	A	G	Met861Val				25.0	
2472–3527	NS1	SNV	2763	T	G	Trp892Gly	15.0	14.3			
		SNV	2950	T	C	Phe954Ser				22.0	
		SNV	3264	A	G	Lys1059Glu			65.3	26.4	43.1
3528–4205	NS2A	SNV	3776	C	T	-			20.6	61.5	
		SNV	3969	G	A	Asp1294Asn			19.3	63.6	10.5
4206–4595	NS2B	SNV	4301	G	A	Met1404Ile			70.9	29.9	32.5
4596–6446	NS3	SNV	4856	C	T	-			67.3	33.3	
		SNV	5662	C	T	Ser1858Phe	47.1	60.9	24.3	60.1	42.5
6447–6827	NS4A	SNV	6651	T	A	Phe2188Ile			59.5	19.6	
		SNV	6651	T	C	Phe2118Leu					29.1
		SNV	6658	T	C	Val2190Ala			21.1	61.1	
		SNV	6756	A	G	Arg2223Gly			10.6	19.4	22.4
		SNV	6771	C	A	Leu2228Ile			23.3		
6897–7649	NS4B	SNV	6971	C	T	-				13.8	
		SNV	7155	T	C	Tyr2356His			89.6	90.3	21.1
7650–10358	NS5	SNV	7792	T	C	Val2568Ala					15.8
		SNV	8133	C	A	Leu2682Ile			11.5		
		SNV	8139	G	A	Val2684Ile			62.9	37.7	38.3
		SNV	8588	C	T	-			10.8	10.5	
		SNV	9690	A	T	Arg3201Trp			69.1	33.3	
	5’UTR	SNV	10484	C	T	3’UTR				16.6	

Note: The position of the sequence variants compared to the reference genome sequence of the PRV_V strain ZIKV retrieved from GenBank (Accession number KX601168).PRV_V was produced originally by Vero cells (inoculum); PRV_H was produced by HEK293 cells acutely infected (four days post infection) by the parental PRV_V strain of ZIKV; Zp_H was produced by the persistently-infected HEK293_Zp cell line. Synonymous nucleotide variations, nucleotide variations or mutations with less than 10% frequency were excluded from the comparison. Allele frequencies higher than 90% are highlighted in red. pi: post-infection.

**Table 2:** Summary of ZIKV RNA genomic variants bearing sequence changes in structural proteins identified in the inoculum FLR ZIKV and ZIKVs produced by acutely and persistently FLR ZIKV-infected HEK293 cells on the structural protein genes in comparison with the reported FLR reference genomic sequence.

Range on Reference Sequence	Encoded Protein	nt Position	Type	Reference Base	Variant	Amino Acid Change	Allele Frequency				
							FLR_V (Inoculum)	FLR_H (4d pi)	Zf_H (8mo pi)	Zf_H (10mo pi)	Zf_H (11mo pi)
457–735	Protein pr	477	SNV	G	A	-			50.31		46.8
		626	SNV	A	G	Asp197Gly		49.57			
		708	SNV	A	G	-				70.0	
		715	SNV	G	A	Glu209Lys			94.38	99.1	97.5
736–960	Membrane glycoprotein M	767	SNV	A	G	Lys226Arg			58.44		65.5
		844	SNV	T	C	Phe252Leu				18.4	29.6
		944	SNV	T	G	Ile285Ser			96.63	27.8	38.7
961–2472	Envelope protein E	1522	SNV	C	T	Leu478Phe		18.06			
		1774	SNV	G	T	Ala562Ser			18.42	100.0	53.8
		1784	SNV	C	T	Ala565Val				67.5	
		1867	SNV	G	A	Val593Met					17.2
2473–3528	NS1	2563	SNV	A	G	Arg825Gly				69.6	
		2945	SNV	A	G	His952Arg			18.52	83.3	17.4
		2999	SNV	C	T	Ser970Leu			10.14		
		3130	SNV	A	G	Met1014Val					35.0
		3157	SNV	C	T	His1023Tyr			25.18		37.2
		3338	SNV	A	G	Glu1083Gly			90.72	100.0	98.9
3529–4206	NS2A	3878	SNV	C	T	Ala1236Val		28.21	96.83	98.4	97.8
		3944	SNV	T	C	Ile1285Thr				60.4	
		4053	SNV	T	C	-					14.0
4207–4596	NS2B	4326	SNV	C	T	-				29.9	
4597–6447	NS3	4857	SNV	C	T	-			14.34	72.2	
		5118	SNV	T	C	-					16.7
		5586	SNV	C	T	-				12.7	43.4
6448–6828	NS4A	6503	SNV	A	G	Glu2138Gly			88.76	100.0	100.0
		6545	SNV	G	A	Arg2152Gln					29.5
		6565	SNV	C	T	Pro2159Ser					16.0
		6659	SNV	T	C	Val2190Ala			13.01	18.0	17.0
		6759	SNV	A	C	Arg2223Ser					25.8
		6759	SNV	A	T	Arg2223Ser				10.9	17.7
6898–7650	NS4B	7156	SNV	T	C	Tyr2356His			98.97	100.0	100.0
		7603	SNV	T	C	Ser2505Pro				64.3	
7651–10359	NS5	7740	SNV	A	G	-			63.29		33.6
		8140	SNV	G	C	Val2684Leu			93.41	99.6	99.1
		8794	SNV	T	C	-		30.73			
		10344	SNV	T	C	-				10.4	42.2

Note: The position of the sequence variants compared to the reference genome sequence of the FLR_V strain ZIKV retrieved from GenBank (Accession number KX087102).FLR_V was produced originally by Vero cells (inoculum); FLR_H was produced by HEK293 cells acutely infected (four days post infection) by the parental FLR_V strain of ZIKV; Zf_H was produced by the persistently-infected HEK293_Zf cell line. Synonymous nucleotide variations, nucleotide variations or mutations with less than 10% frequency were excluded from the comparison. Allele frequencies higher than 90% are highlighted in red. pi: post-infection.

## Abbreviations

ZIKV	Zika virus
HEK293	Human embryonic kidney
MNV	West Nile Virus
CNS	central nervous system
PRV	Puerto Rico
FLR	Colombia
CPE	cytopathic effects
